# Solid Pseudopapillary Neoplasm of the Uncinate Process of the Pancreas: A Case Report and Review of the Literature

**DOI:** 10.7759/cureus.15125

**Published:** 2021-05-19

**Authors:** Muhammad Rafay Khan Niazi, Samyak Dhruv, Abhishek Polavarapu, Mesut Toprak, Indraneil Mukherjee

**Affiliations:** 1 Internal Medicine, Staten Island University Hospital | Northwell Health, Staten Island, USA; 2 Internal Medicine, Northwell Health, New York, USA; 3 Gastroenterology, Staten Island University Hospital | Northwell Health, Staten Island, USA; 4 Pathology and Laboratory Medicine, Staten Island University Hospital | Northwell Health, Staten Island, USA; 5 Surgery, Staten Island University Hospital | Northwell Health, Staten Island, USA

**Keywords:** pancreatic tumors, solid pseudopapillary neoplasm, diagnosis of rare cases, young female with abdominal pain, epithelial tumor

## Abstract

Solid pseudopapillary neoplasm (SPN) is a rare pancreatic neoplasm that accounts for 1-3% of all pancreatic tumors. SPNs are most commonly found in females in their third and fourth decades of life. Even though the majority of the tumors are benign, malignant tumors have also been reported. Given its rare occurrence, it remains a clinical dilemma in gastroenterology, oncology, and pathology. It is critical to diagnose it early and differentiate it from other similar pancreatic tumors or cysts to ensure favorable patient outcomes. Advanced imaging techniques, characteristic histologic findings, and immunohistochemical analysis can help in diagnosing solid pseudopapillary tumors. Early diagnosis and surgical resection can result in a cure in most cases, and tumor recurrence is extremely rare. In this report, we present a case of a 40-year-old female patient who presented to the emergency room and was diagnosed with SPN of the pancreas.

## Introduction

Solid pseudopapillary neoplasm (SPN) of the pancreas was first described by V.K. Frantz in 1959. It mainly affects women, usually in the third and fourth decades of life, and is associated with a good prognosis: a 97% five-year survival rate [1-2]. Although most of the SPNs are benign, 10-15% of them turn out to be malignant [1]. It is located in the body or tail of the pancreas in more than half of the cases, while in the remaining instances, it is found in the head of the pancreas [1]. There are scarce data in the literature about this condition, and hence its diagnosis and management can be challenging. We present a rare case of SPN in a 40-year-old female who initially presented to the emergency department (ED) with the complaint of intermittent abdominal pain that had started a day ago. Imaging revealed a mass in the uncinate process of the pancreas, which was later diagnosed as SPN. This case report also includes a brief review of literature that highlights the importance of early diagnosis and proper management of these rare cases.

## Case presentation

**Figure 1:**
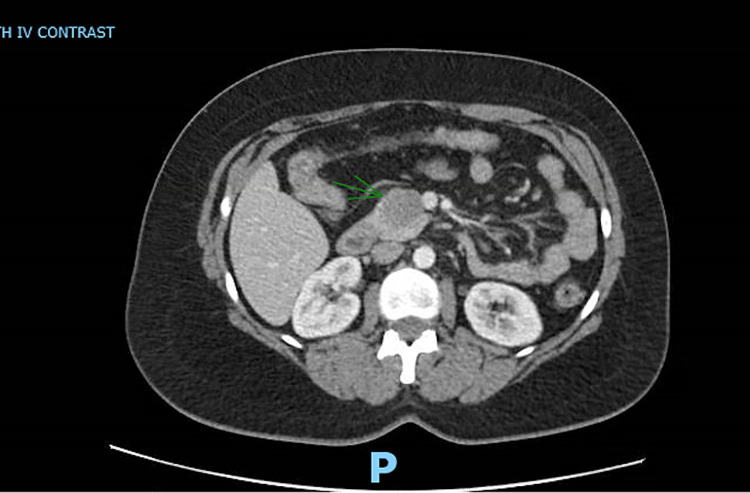
CT scan of the abdomen with intravenous contrast An axial section image of CT abdomen with intravenous contrast showing a 3.6-cm well-demarcated lobular hypoattenuating mass in the uncinate process of the pancreas (arrow) CT: computed tomography

**Figure 2:**
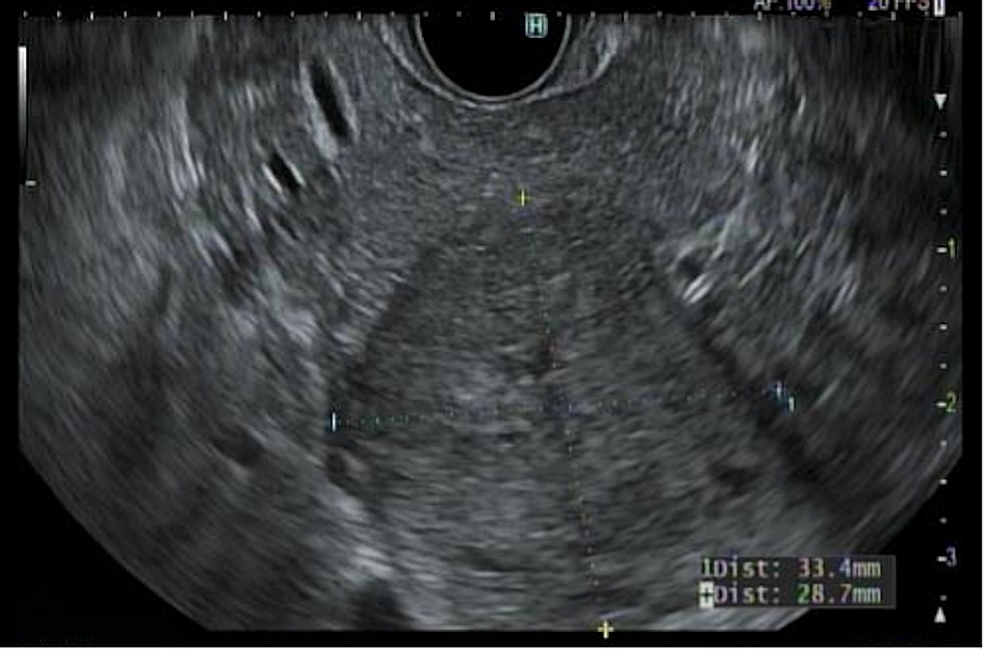
Image from endoscopic ultrasonography A linear echoendoscope (or endoscopic ultrasonographic) image demonstrating a 3.3 x 2.9-cm well-circumscribed isodense lesion in the uncinate process of the pancreas

**Figure 3:**
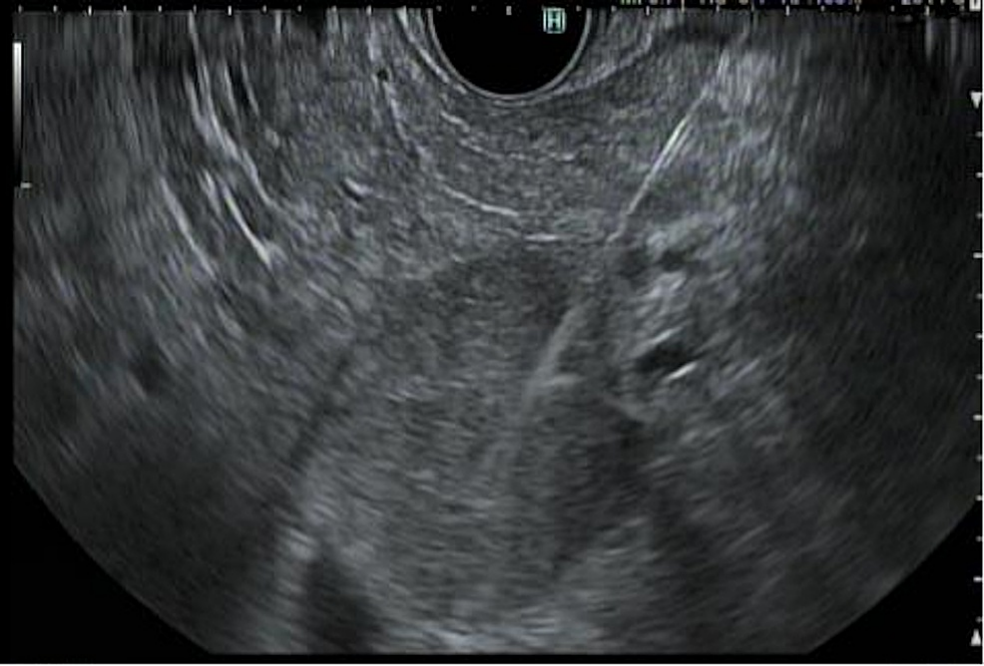
Image of EUS-guided fine-needle biopsy EUS-guided fine-needle biopsy of the pancreatic mass using a 22-gauge needle under Doppler guidance to avoid vascular interference EUS: endoscopic ultrasonography

A 40-year-old female with a past medical history of uterine polyp presented to the emergency room with epigastric pain. She endorsed the pain to be waxing and waning, non-radiating, and 8/10 in severity, which had started a day before the presentation. She denied any fever, chills, dysuria, nausea, or vomiting. Her vital signs were within normal limits. Her routine labs were also normal [aspartate aminotransferase (AST): 13 IU/L; alanine aminotransferase (ALT): 13 IU/L; alkaline phosphatase (ALP): 67 IU/L; total serum bilirubin: 0.3 mg/dl; and serum lipase: 35 U/L]. CT scan of the abdomen was performed, which showed a 3.6-cm lobulated mass in the uncinate process of the pancreas (Figure 1). MRI of the abdomen was also performed with the intravenous (IV) contrast, which showed a smoothly marginated 3.6-cm lobular soft tissue mass within the pancreatic uncinate process. There was no evidence of pancreatic or biliary ductal dilatation and vascular involvement. These findings raised the suspicion for solid pseudopapillary epithelial neoplasm (SPEN). Serum carbohydrate antigen 19-9 (CA19-9) was checked, and it came back normal (2 U/ml). The gastroenterology team was consulted and the patient underwent endoscopic ultrasonography (EUS) with biopsy of the uncinate process the following morning (Figures 2, 3).

**Figure 4:**
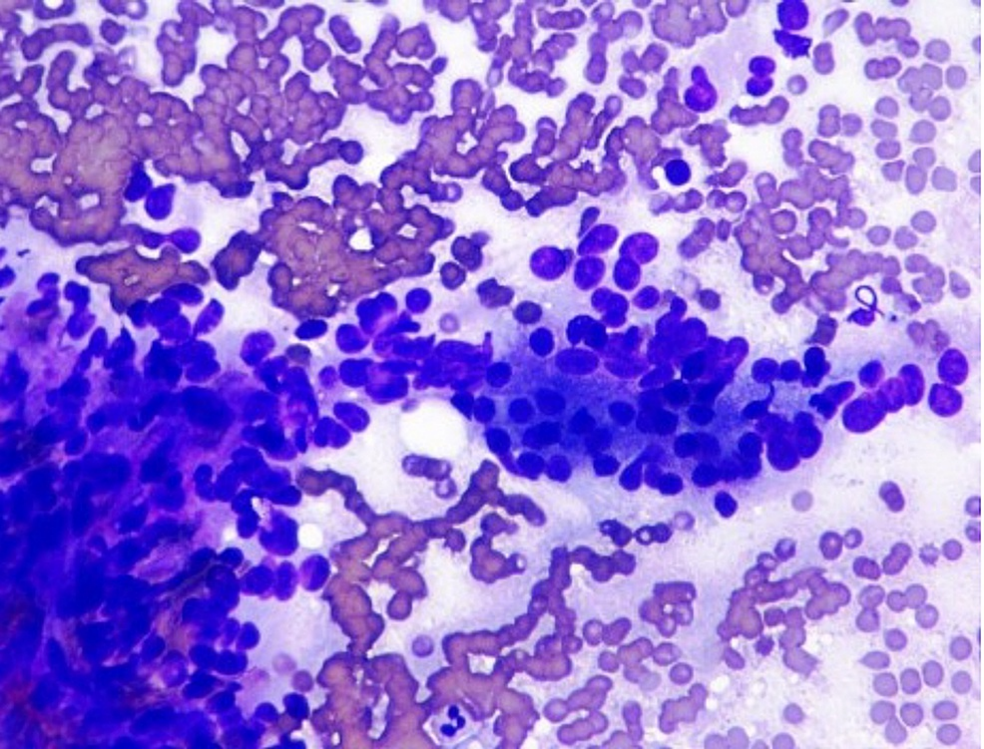
Tissue cytology Cellular smears composed of small to medium-sized cells surrounding the fibrovascular cores with abundant myxoid material. The cells have round to oval nuclei, inconspicuous nucleoli, occasional nuclear grooves, and granular cytoplasm (Diff-Quik stain)

**Figure 5:**
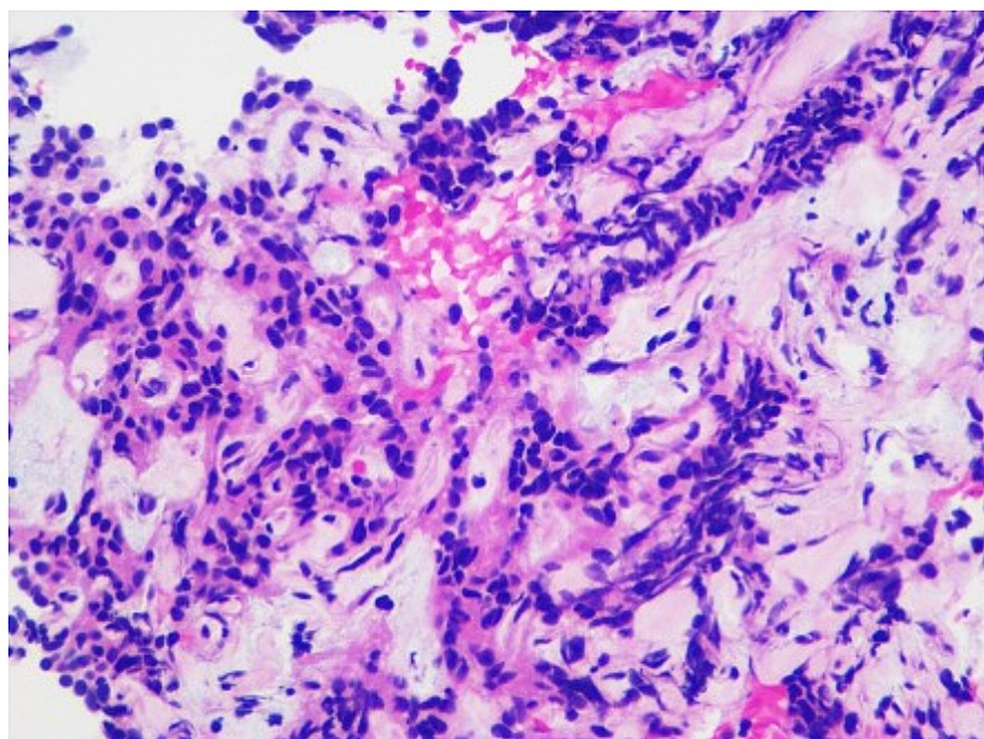
Tissue cytology (H&E stain) Cell blocks display cells with round to oval nuclei and mild anisonucleosis, adjacent to amorphous, myxoid, and hyaline material (H&E stain)

**Figure 6:**
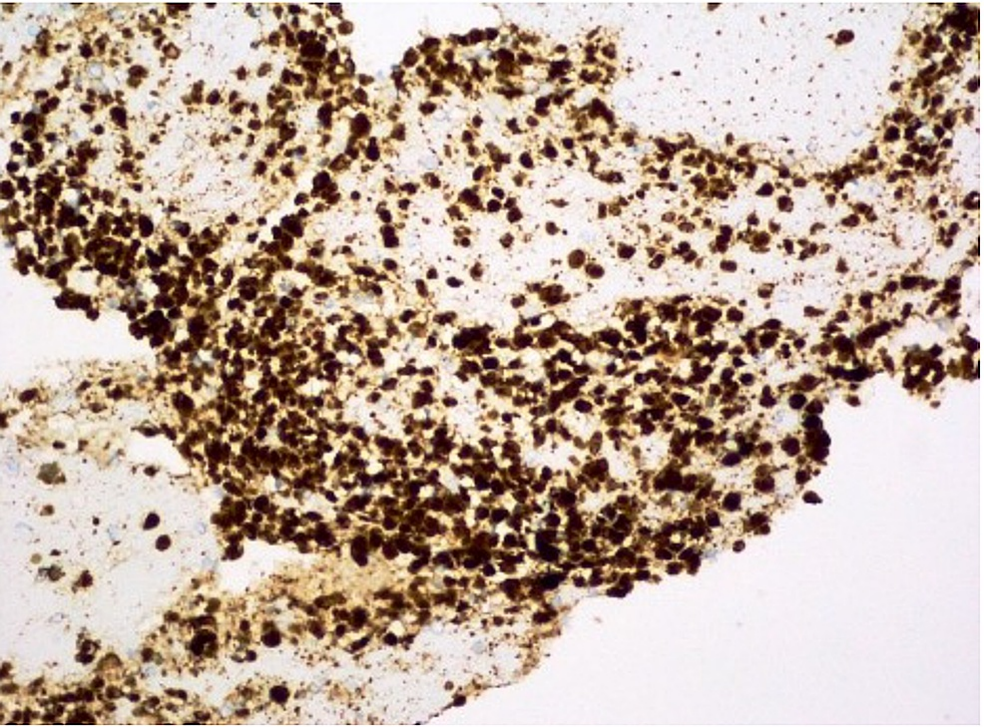
Beta-catenin stain is found strongly positive

Biopsy tissue was sent for histology and immunohistochemical analysis. Cellular smears were composed of small to medium-sized cells found as singly, loosely cohesive sheets, and small clusters. The cells had a plasmacytoid appearance with round to oval nuclei, inconspicuous nucleoli, occasional nuclear grooves, and granular cytoplasm (Figure 4). Cell blocks displayed cells with round to oval nuclei and mild anisonucleosis adjacent to amorphous myxoid and hyaline material (Figure 5). Immunohistochemical studies showed positive beta-catenin (Figure 6), alpha-1-antitrypsin (focal), alpha-1-antichymotrypsin, synaptophysin (few cells), chromogranin (few cells), p16 (focal), vimentin, Cam 5.2, AE1/AE3, cluster of differentiation 56 (CD56), CD10, progesterone receptor (PR), and E-cadherin, while negative CD117 and CK19. The cytomorphology in conjunction with immune profile and imaging findings favored a diagnosis of SPN over pancreatic neuroendocrine or other neoplasms. The patient's pain gradually improved, and she was discharged with the outpatient follow-up with the surgery team for the resection of the tumor. She did not follow up after that.

## Discussion

SPN is a rare exocrine tumor of the pancreas that accounts for 1-3% of all pancreatic neoplasms [3]. It is a low-grade, malignant epithelial tumor with uncertain differentiation. According to the WHO classification, SPNs with malignant characteristics such as vascular and nerve sheath invasion, lymph node, or liver metastasis are designated as solid pseudopapillary carcinomas. It mainly affects young women in the third and fourth decade of their lives (female to male ratio: 10:1). In men, it tends to occur at an older age with a more aggressive clinical course [4]. There is a predilection for African American and Asian women and it is rarely seen in children. The most common location is the pancreas’ body or tail; however, it has been reported in the extra-pancreatic tissues as well [5]. Macroscopically, it appears as a small, encapsulated mass with areas of hemorrhage, necrosis, and calcifications. In the majority of the cases, patients present with abdominal pain. However, it may present as gradually enlarging abdominal mass in some patients. In very rare cases, the tumor is discovered after a complication with intertumoral hemorrhage or intraperitoneal rupture. The differential diagnosis of SPN includes pancreatic endocrine neoplasm, acinar cell carcinoma, well-differentiated ductal cell carcinoma, pancreatic mucinous neoplasm, and pseudocyst. The pathogenesis of SPN remains unclear and there are various theories behind its origin. Two basic theories behind its origin are as follows: (1) origin from genital ridge-related cells and (2) origin from pancreatic progenitor cells. A notable majority of the cells are found to have a beta-catenin coding gene mutation (CTNNB1) [6]. These mutations can affect the Wnt signaling pathways and self-renewal capacity of stem cells. SPN cells are reported to be positive for beta-catenin, alpha-1-antitrypsin, vimentin, CD10, CD56, and PRs by immunohistochemical analysis. However, these staining patterns do not correlate between these defined cell lineages of the pancreas and SPN. As a result, it is speculated that SPN cells show multipotent differentiation potential [7]. Other authors have suggested an interplay of hormones as supported by the disease's predominance in women and the presence of progesterone receptors in these tumors.

Histologically, these tumors are very similar to other pancreatic cysts or tumors. It is often difficult for a pathologist to diagnose them based on histology and cytology alone. However, certain cellular features like hyaline globules, nuclear grooving, pseudopapillary, and foamy histiocytes, favor a diagnosis of SPN [8]. Most investigators recommend an immunohistochemistry panel to differentiate among these entities. Immunohistochemistry reveals diffuse a-antitrypsin (+), antichymotrypsin (+), vimentin (+), synaptophysin (+) (few cases), cytokeratin (AE1/AE3) weakly, CD10 (+), CD56 (+), PR (+), and chromogranin A Omni (-) [9]. However, after analyzing 50 cases, Pettinato et al. [10] stated that fine-needle aspirate cytological features are highly characteristic of SPN and make it distinctive from other pancreatic tumors.

In the past, the diagnosis was rarely made preoperatively. Currently, with the advancement in imaging, diagnosis can be supported radiologically. Usually, it is seen as a well-defined tumor, poorly vascularized with mixed composition associated with solid and cystic areas giving it a heterogenous appearance on ultrasound. CT and MRI reveal a fibrous capsule or subjugation into neighboring tissues without invading any organs with hemorrhagic foci [11]. MRI is usually superior to CT due to its superior contrast resolution. Hemorrhagic content appears as high-intensity signal areas on MRI. In contrast, the capsule is identified as a thin hypointense rim. Angiography reveals an avascular or hypovascular mass and helps to distinguish it from the neighboring structures. Several studies endorse the use of preoperative EUS-guided fine-needle biopsy of the tumor for preoperative detection of the tumor [12]. SPNs are usually not associated with any specific clinical laboratory findings or tumor makers.

Overall, SPNs have a good prognosis with local resection leading to complete recovery. The eight-year survival rate is 85% in these patients after local resection. This is comparable to the survival rate in patients with metastatic disease after local resection [13]. The site of resection depends on the location of the tumor. Lesions in the head are treated with pancreatoduodenectomy (Whipple procedure). In contrast, those located in the tail or the body are treated with distal pancreatectomy. Poor prognostic factors for disease recurrence include (1) tumor size of >8 cm, (2) perineural invasion, (3) vascular invasion, (4) cellular atypia, (5) systemic metastasis, and (6) peritoneal seeding [14]. Recurrence is very infrequent, with <2% mortality [15]. Another important prognostic marker is the Ki-67 index, a pathologic marker for proliferation. It is believed that a high Ki-67 index represents increased mitotic activity and thus indicates a more aggressive tumor. Several studies have linked elevated Ki-67 with poorer disease-specific and disease-free survival [14]. Metastatic disease is uncommon, occurring only in <10% of cases. The most common sites of metastasis are loco-regional lymph nodes and the liver. For metastatic tumors, a debulking surgery is recommended against the principles of management of other pancreatic malignancies. It should be noted here that the biopsy of the cyst or its rupture during the surgery can seed the peritoneum, leading to disease recurrence in the peritoneum [16]. For unresectable tumors, radiotherapy is recommended, as these tumors are seen to be radiosensitive [17]. The data relating to the use of chemotherapy in these tumors with or without metastasis is sparse.

There are case series about the diagnosis of SPN in pregnant females due to dramatic enlargement of tumors during pregnancy. Female patients with strong immunoreactivity for progesterone receptors can be particularly vulnerable to increased tumor growth during pregnancy secondary to higher hormone levels. Again, the preferred treatment remains the same with local resection in this population as well, preferably in the second trimester, since fetal organomegaly is complete by then. The size of the fetus at this time may allow for an easier surgical procedure compared to third-trimester operations [18].

## Conclusions

SPN is a rare pancreatic tumor with an excellent prognosis and survival rate. Even though its diagnosis remains a challenge, proficient use of imaging modalities, histology, and immunohistochemistry can help differentiate it from other malignant pancreatic tumors. Although resection is curative in most cases, close follow-up is needed for the early diagnosis of local recurrence. It is important for clinicians to be wary of this entity and include it in their differentials of the pancreatic mass.
